# A high‐performance method for quantitation of aflatoxins B1, B2, G1, G2: Full validation for raisin, peanut matrices, and survey of related products at Ho Chi Minh City

**DOI:** 10.1002/fsn3.3594

**Published:** 2023-08-15

**Authors:** Thanh Duy Nguyen, Thuy Ngan Ha Nguyen, Tuan Kiet Ly, Quoc Hung Nguyen, Thanh Tho Le, Van Hai Chu, Tien Dat Nguyen, Dinh Vu Le

**Affiliations:** ^1^ Graduate University of Science and Technology, VietNam Academy of Science and Technology (VAST) Hanoi Vietnam; ^2^ Center of Analytical Services and Experimentation HCMc Ho Chi Minh Vietnam; ^3^ Department of Science and Technology of Ho Chi Minh City Ho Chi Minh Vietnam; ^4^ Center for Research and Technology Transfer, VAST Hanoi Vietnam; ^5^ Faculty of Chemical Engineering, Industrial University of Ho Chi Minh City Ho Chi Minh Vietnam

**Keywords:** aflatoxin, derivatization, fluorescence detection, liquid chromatography‐mass spectrometry, post‐column, UPLC

## Abstract

Optimization and validation for simultaneous quantitation of four aflatoxins B1, B2, G1, and G2 in peanuts and raisins were performed on ultra‐performance liquid chromatography in a combination of fluorescence detector, without derivatization. The advantages were short analysis time, simple sample handling, and reduced solvent consumption. Instrument detection limits of AFB1, AFB2, AFG1, and AFG2 were 0.07, 0.01, 0.1, and 0.008 μg/kg, respectively, lower than those obtained by LCMSMS and HPLC‐FLD with derivatization. Two solvent mixtures were chosen for two different matrices whose matrix effect was not negligible (2.81%–8.04% for peanuts and 5.63%–11.43% for raisins). The linear ranges were from 0.2 to 20 μg/L for AFB1 and AFG1 and from 0.05 to 5 μg/L for AFB2 and AFG2. The limits of detection and quantification were 0.025–0.1 and 0.075–0.3 μg/kg for peanuts and raisins, respectively. Recoveries at three other concentrations from 0.75 to 125 μg/kg of total aflatoxins were obtained between 76.5% and 99.8% (with RSD < 6%) following the *SANTE 11312/2021*. Validation parameters complied with the requirements of ISO/IEC 17025:2017. The extracts and the sample could be stabilized at 4°C and 20°C for 24 h and at −20°C for up to 21 days, respectively. Thus, the study can be used as a standard method for the analysis of Aflatoxins (AFs) in peanut and raisin matrices. Investigation of 350 peanut samples collected at Markets in the central districts of HCM city showed that 28.6% were contaminated with AFB1 from 0.31 up to 554 μg/kg; 13.4% contained AFB2, and 5.7% of AFG1 in the range of 0.4–53 μg/kg and 0.4–9.57 μg/kg, respectively; AFG2 (about 0.6%) was detected from 0.45 to 0.75 μg/kg. Meanwhile, 12.8% exceeded the total aflatoxins limit, and 13.4% exceeded the AFB1 limit. AFs were almost not found in the 350 raisin samples.

## Graphical abstract

Optimization and validation for simultaneous quantitation of four aflatoxins B1, B2, G1, and G2 in peanuts and raisins were performed on ultra‐performance liquid chromatography in a combination of fluorescence detector, without derivatization. And investigations of 350 peanut and raisin samples collected at Markets in the central districts of HCM city were done.

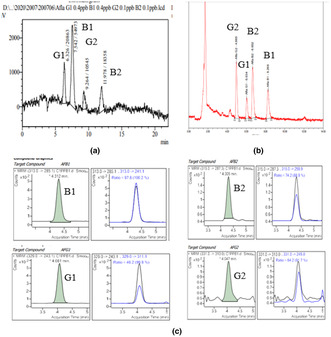

## 1 INTRODUCTION

Aspergillus fungi are well known for producing Aflatoxin (AF), which can harm the physiological status of humans and animals by causing DNA damage, cancer, and developmental abnormalities in embryos with long‐term exposure. When consumed, carbonyl and methylene‐intercalated groups, known as polyketides, are absorbed, transformed, and delivered to different body parts. Long‐term exposure to AF can lead to aflatoxicosis, an acute poisoning that can be life‐threatening and primarily causes liver damage. Research has shown that children are most exposed to all foods contaminated with AF, followed by adolescents and adults, who are the least exposed group. Available evidence suggests that infants and young children are more likely to experience the harmful effects of mycotoxins due to their immature metabolism and higher absorption‐to‐body weight ratio, higher metabolic rate, and lower detoxification capacity than adults. Four common types of AF are aflatoxin B1 (AFB1), aflatoxin B2 (AFB2), aflatoxin G1 (AFG1), and aflatoxin G2 (AFG2). Based on epidemiological studies, AFB1 is the deadliest AF. In addition to the impact on human health, AF also leads to significant economic losses due to the widespread contamination of food. Therefore, detecting and quantifying AF in food are essential to ensure safety (Shabeer et al., 2022).

Aspergillus fungi can colonize agricultural products prior to and during harvest, as well as during storage if unsuitable conditions exist. The hot and humid climate in Vietnam provides favorable conditions for mold growth. Therefore, these fungi are commonly detected in crops such as wheat, rice, corn, peanuts, oilseeds, and other harvested commodities. Raisins are rich in nutrients and energy, and peanuts are an agricultural product with a reasonably high lipid content, an oil‐loving type used quite a lot in the food processing industry. In addition, raisins and peanuts are within the maximum allowable group of products according to Vietnam and international standards. However, the presence and extent of mycotoxin contamination in these two food items have not been thoroughly researched and surveyed in Ho Chi Minh City. Consequently, raisins and peanuts have been selected as the focal products in this study to address this research gap (Ajmal et al., 2022).

Due to the detrimental effects of AFs on human health, the allowable limits of these toxins in food are set very low. Maximum limits (ML) of aflatoxins in food have been established by Vietnam and other countries, and Table 1 provides an overview of these limits (Food Safety and Standards Authority of India [FSSAI], 2016; The National Technical Regulation QCVN 8‐1:2011,BYT, 2011).

**TABLE 1:** Comparison of maximum limit (ML) of AF for peanuts and raisins in different countries.

Compound	Matrix	ML (μg/kg)				
		EU	USA	China	Japan	VN
AFB1	Peanuts	2	—	5	—	2
Total AF		4	20	—	10	4
AFB1	Raisins	5	—	5	—	5
Total AF		10	20	—	10	10

Abbreviation: —, not applicable.

Many methods for analyzing AF depending on sample matrices have been reported in previous studies (AOAC Official Method 994.08, 1997; AOAC Official Method 2005.08, 2005; Benvenuti & Di Gioia, 2009; Bessaire et al., 2019; Deng et al., 2020; Food Safety and Standards Authority of India [FSSAI], 2016; Lv et al., 2020; Ouakhssase et al., 2019; The National standard TCVN 7930: 2008, 2008; The National standard TCVN 9522:2012, 2012). The thin‐layer chromatography and Elisa kit could identify total aflatoxins with a low sensitivity or often give a false positive (Food Safety and Standards Authority of India [FSSAI], 2016). Some techniques were also reported for the determination of the total of AF or single AFB1, such as high‐performance liquid chromatography coupled with fluorescence detection (HPLC‐FD) with pre‐column or post‐column derivatization (The National Standard TCVN 7930: 2008, 2008; The National Standard TCVN 7930: 2012, 2012). Currently, HPLC‐FD has been published for the simultaneous quantitation of AFB1, AFB2, AFG1, and AFG2 in peanuts according to AOAC method 994.08 with trifluoroacetic acid pre‐column derivatization (AOAC Official Method 994.08, 1997) or AOAC method 2005.08 with post‐column photochemical derivatization which is expensive equipment (AOAC Official Method 2005.08, 2005). The derivatization technique required toxic chemicals that also risk human health and the environment. The technique without derivatization was also published using an HPLC‐FD system that had a poor sensitivity (Benvenuti & Di Gioia, 2009) or using liquid chromatography coupled to a mass spectrometry system (LC‐MS/MS) which was very costly equipment (Bessaire et al., 2019; Deng et al., 2020; Lv et al., 2020; Ouakhssase et al., 2019).

To the best of our knowledge, there is a dearth of published research both in Vietnam and worldwide on the simultaneous analysis of AFB1, AFG1, AFB2, and AFG2 in peanuts and raisins using ultra‐performance liquid chromatography coupled with a special fluorescence detector that does not require derivatization.

In this study, we aimed to optimize and validate the simultaneous analysis of AFB1, AFB2, AFG1, and AFG2 on an ultra‐performance liquid chromatography system in combination with fluorescence detection (UPLC‐FD) without pre‐column or post‐column derivatization on raisins and peanuts. Effects of two different matrices on the quantitation of aflatoxins were considered. Validation was entirely performed to obtain parameters of the ISO/IEC 17025:2017 (The National standard TCVN ISO/IEC 17025:2017, 2017) requirements. In addition, investigate some actual samples collected from local markets in Ho Chi Minh City, Vietnam.

## 2 EXPERIMENTATION

### 2.1 Chemicals

Phosphate‐buffered saline pH 7.4 (PBS), distilled water (H_2_O), formic acid (HCOOH), methanol (MeOH), and acetonitrile (ACN) for HPLC grade were purchased from Merck. In addition, immunoaffinity column 3 mL (IAC‐ AflaTest WB) was bought from VICAM.

Total aflatoxins were 5.0 μg/mL in ACN containing 2.0 μg/mL of AFB1, 2.0 μg/mL of AFG1, 0.5 μg/mL of AFB2, and 0.5 μg/mL of AFG2 which were supplied by Dr. Ehrenstorfer GmbH brand, Germany. The stock standard solution was stored in an amber flask at −20°C for up to 12 months. Intermediate solutions were prepared by dilution in ACN and used for at least 6 months.

### 2.2 Preparation of calibration curve

Prepare different concentrations of AF from intermediate standard solution. Use a solution of ACN/ H2O with 0.2% of HCOOH (1/1, v/v) to obtain calibration from 0.05 to 20 μg/L for raisins and peanuts.

### 2.3 Sample preparation

Take 5000 g of homogenized sample into a centrifuge tube. Add 20 mL of ACN/H_2_O (60/40, v/v) for peanut and MeOH/H_2_O (80/20, v/v) for raisin. Extraction was performed under ultrasound for 30 min, centrifuged at 9000 rpm for 3 min, and filtered through Ω 110 mm filter paper. Take 5 mL of the filtrate, and add 20 mL of distilled water (for peanut matrix) and 20 mL of PBS buffer (for raisin matrix). Vortex for 1 min, centrifuge at 9000 rpm for 3 min. Add 20 mL of the solution after centrifugation to the IAC. Wash impurities with 20 mL of distilled water. Elute with 3 mL of MeOH, dry under nitrogen flow, and add 1.0 mL of ACN/H_2_O with 0.2% of HCOOH (1/1), vortex. Filter through a 0.45 μm PTFE filter before injection into the ultra‐performance liquid chromatography coupled with fluorescence detection (UPLC‐FD) system.

### 2.4 Equipment

The H‐Class UPLC with a fluorescence detector system from waters was used with a BEH C18 reversed‐phase column (1.7 μm × 2.1 mm × 150 mm) and a guard column. The mobile phase was 0.1% HCOOH in H_2_O/ACN/MeOH (64/18/18 in volume), with a flow rate of 0.2 mL/min, column temperature of 40°C, and injection volume of 5 μL. Fluorescence detector parameters were excitation wavelength Ex = 365 nm and emission wavelength Em = 455 nm.

The HPLC with a fluorescence detector system from Shimadzu was connected with an Altima C18 reversed‐phase column (5 μm × 4.6 mm × 250 mm) and a guard column. The mobile phase was ACN/MeOH/H_2_O (46/38/16, v/v/v), with a flow rate of 0.7 mL/min, ambient temperature, and injection volume of 20 μL. Fluorescence detector parameters were excitation wavelength Ex = 360 nm and emission wavelength Em = 440 nm.

The LC/MS/MS from Agilent was combined with an XDB‐C18 reversed‐phase column (5 μm × 4.6 mm × 150 mm) and a guard column. The mobile phase was isotactic mobile phase A (ACN/CH_3_COONH_4_ (1/9)) and mobile phase B (MeOH), and mobile phase A/mobile phase B ratio (8/2), with a flow rate of 0.3 mL/min, ambient temperature, and injection volume of 5 μL.

## 3 RESULT AND DISCUSSION

### 3.1 Comparison of different analytical techniques

**FIGURE 1:**
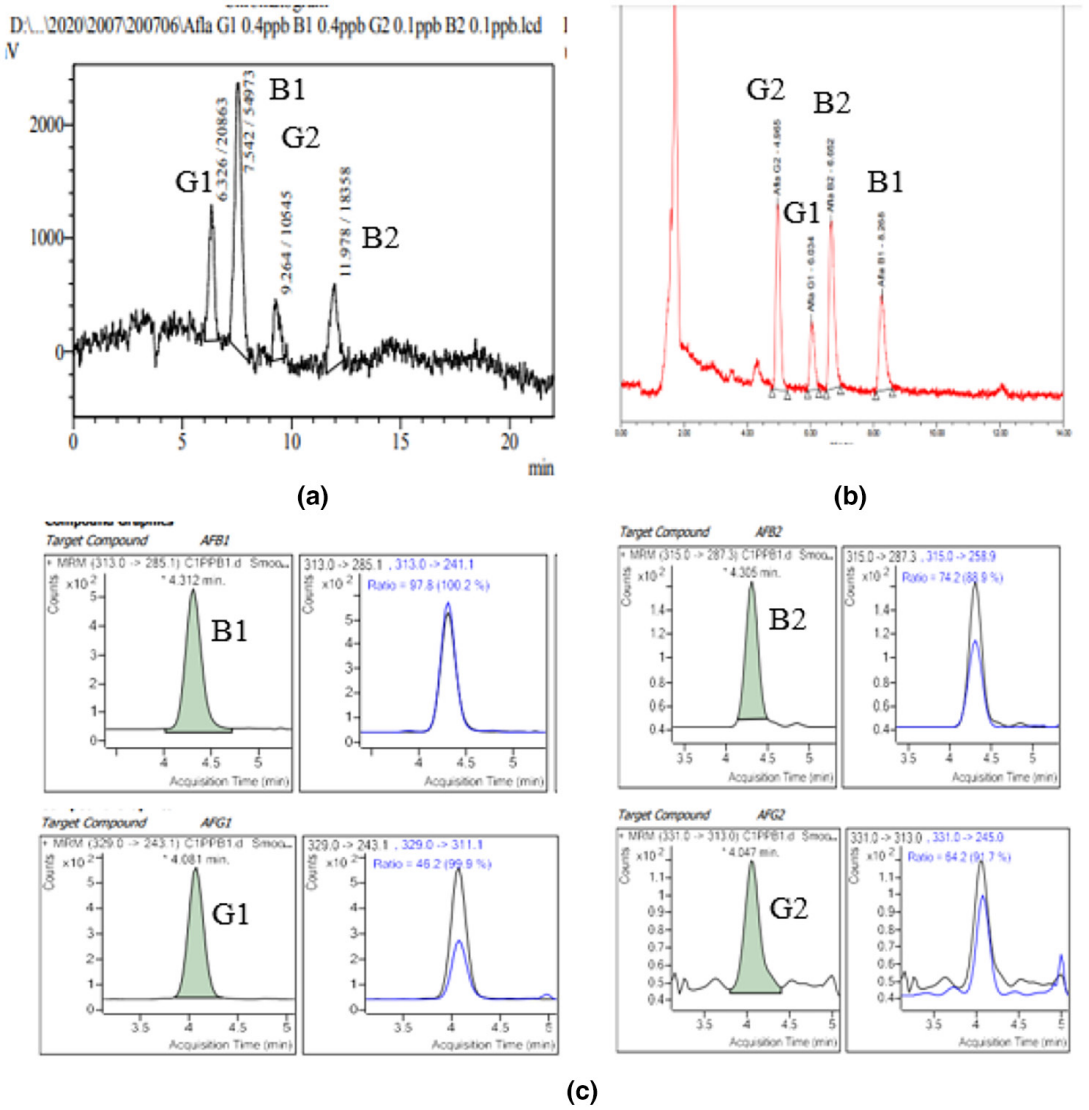
Chromatograms of Aflatoxins B1, B2, G1, and G2 for three methods including HPLC‐FD with pre‐column derivatization (a), UPLC‐FD without derivatization (b), and LC/MS/MS (c).

Two techniques HPLC‐FD with pre‐column derivatization with trifluoroacetic acid (ISO 16050:2011) (EN ISO 16050:2011, 2011) and LC/MS/MS (FDA) (Liao et al., 2011) were selected to compare with UPLC‐FD without derivatization. The evaluation criteria are based on the instrument's detection limit at the same standard concentration. Select a concentration of 1 μg/L total aflatoxin (0.4, 0.1, 0.4, 0.1 for AFB1, AFB2, AFG1, and AFG2, respectively) by the limit specified by QCVN 8:1/2011/BYT Vietnam (The National Technical Regulation QCVN 8‐1:2011/BYT, 2011). Figure 1 shows the chromatograms of AFB1, AFB2, AFG1, and AFG2 obtained by three different techniques.

The chromatogram profile obtained by HPLC‐FLD (Figure 1a) showed a more significant baseline than that of UPLC‐FLD, even though 20 μL of the sample was injected. The order of retention times of AFs B1, B2, G1, and G2 is also different between HPLC‐FLD and UPLC‐FLD (or LC/MS/MS) (Figure 1b,c) due to derived or non‐derived aflatoxins. The chromatogram from the UPLC‐FLD technique also showed sharp peaks and a high intensity in comparison to the other. Separation of peaks in the HPLC and UPLC was better than LC.

The UPLC is based on the principle that the particle size of stationary phase is less than 2 μm. While in the HPLC, the former is 3–5 μm. Particle size significantly impacts the analyte band as it relates to the eddy diffusion. The path that analyte molecules take to transfer from the bulk mobile phase to the surface of the particle and around those particles takes less time as the particle size is decreased. Larger particles cause analyte molecules to travel longer, more indirect paths (Suryavanshi et al., 2021). In this study, UPLC BEH C18 column is packed with 1.7 μm particles; HPLC‐MS C18 column is packed with 5 μm particles; and HPLC C18 column is packed with 5 μm particles. Choosing UPLC with BEH C18 column leads to a significant reduction in analysis time and solvent consumption as well. Particularly, the Waters ACQUITY UPLC‐FLD is designed with a large flow cell and a mercury‐xenon lamp that provide a necessary sensitivity for quantitation of AF without derivatization (Benvenuti & Di Gioia, 2009).

The instrument detection limit (iDL) of AFB1, AFB2, AFG1, and AFG2 achieved from UPLC‐FLD was in the range of 0.008–0.092 μg/L, which is lower than that of LCMSMS (in the technique range of 0.05–0.24 μg/L) and of HPLC‐FLD (in the range of 0.071–0.12 μg/L; Table 2). Based on IDL, injection volume, mobile phase flow, and separation of peaks, the UPLC‐FLD technique showed more advantages than HPLC‐FLD and LC/MS/MS techniques.

**TABLE 2:** Comparison of IDL calculated from the three other techniques with iDL = 3 × *C*_min_/(S/N).

AF concentration (μg/L)		HPLC‐FLD with derivatization			UPLC‐FLD without derivatization			LC/MS/MS without derivatization		
		RT (min)	S/N (*n* = 3)	iDL (μg/L)	RT (min)	S/N (*n* = 3)	iDL (μg/L)	RT (min)	S/N (*n* = 3)	iDL (μg/L)
AFB1	0.4	7.41	17	0.071	8.261	16	0.075	4.312	4	0.300
AFB2	0.1	11.61	4	0.075	6.675	30	0.010	4.306	6	0.050
AFG1	0.4	6.24	10	0.120	6.055	13	0.092	4.061	5	0.240
AFG2	0.1	9.09	3	0.100	4.977	39	0.008	4.047	6	0.050

### 3.2 Influence of extraction solvent on different matrixes

Aflatoxins have good solubility in a polar solvent. Many studies showed that efficient extraction was carried out with organic‐aqueous solvents (Agriopoulou et al., 2020). Thus, this study used two extraction solvents, including acetonitrile/water (6:4, v/v) and methanol/water (8/2, v/v). Standards were spiked into samples at three other concentrations of ML, 5 × ML, and 10 × ML with ML as the ML of aflatoxin B1 (2 μg/kg for peanut and 5 μg/kg for raisin) according to Commission Regulation (EC) No 1881/2006 (Commission Regulation (EC) No 1881/2006, 2006). Then, the samples were prepared as mentioned above.

**FIGURE 2:**
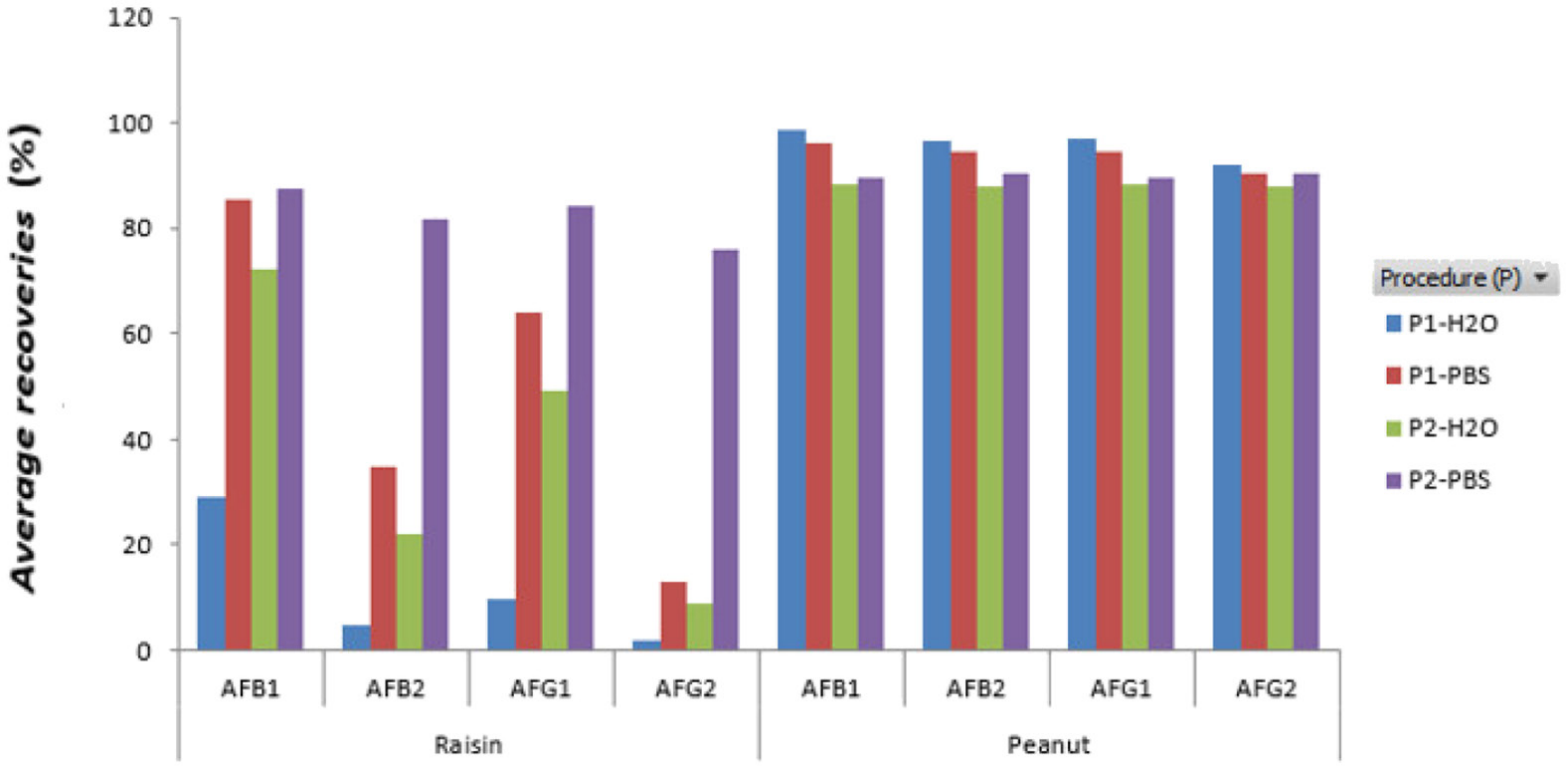
The extraction efficiency of aflatoxins B1, B2, G1, and G2 in peanut and raisin samples with different extraction conditions.

The results in Table 2 showed that using extraction solvent MeOH/H_2_O (8/2, v/v) and diluting the filtrate with PBS before loading the IAC column could be a suitable choice for the peanut matrix. Extraction efficiency was the highest, with a recovery range from 92% to 99% (except for AFG2 at a 10 × ML concentration of about 86%). The ACN/H_2_O (6/4, v/v) solvent could be chosen for the raisin matrix. The filtrate was diluted with H_2_O before passing through the IAC column. The recovery was obtained from 79% to 95% (except for AFG2 at 5 × ML and 10 × ML concentration about 73%). The relative extraction efficiencies of different procedures were intuitively illustrated by the column chart in Figure 2.

The recovery study aimed to evaluate the analytical steps after the extraction, mostly the efficiency of the immunoaffinity column using different solvents and the accuracy of the HPLC analysis. As a result (Table 3), the recovery values for the other extraction solvents were very uniform and showed ACN/H_2_O (6/4), at a range percentage of 92.6%–99.2%, was suitable for fat food (peanuts), and MeOH/H_2_O (8/2), at a range percentage of 78.7%–99.4%, was ideal for carbohydrate food (raisins). Therefore, the extraction efficiency of aflatoxins in raisins by acetonitrile–water is less than that of methanol–water. PBS buffered increased the extraction efficiency for both acetonitrile–water and methanol–water. For peanut matrix with low carbohydrate content, the aflatoxins recovery was very high, and there was almost no significant difference between the two solvent systems. The results are promising for research application development to extract aflatoxin in peanuts with a mixture of acetonitrile/water 60/40 (v/v) from Romer labs (Romer, 2007), and a combination of methanol/water 80/20 (v/v) for raisin from Food Additives & Contaminants journal (Asghar et al., 2016).

**TABLE 3:** Average recoveries (%) and relative standard derivation (RSD %, in parenthesis) obtained with different extraction solvents in peanuts and raisins spiked at three concentration levels ML, 5 × ML, 10 × ML (*n* = 6 for each level).

	Acetonitrile–water (6:4, v/v)‐DL water (*n* = 6) (P1‐H_2_O)		Acetonitrile–water (6:4, v/v)‐ PBS buffered (*n* = 6) (P1‐PBS)		Methanol–water (8:2, v/v)‐DL water (*n* = 6) (P2‐H_2_O)		Methanol–water (8:2, v/v)‐ PBS buffered (*n* = 6) (P2‐PBS)	
	% recovery (RSD %)							
	Raisins	Peanuts	Raisins	Peanuts	Raisins	Peanuts	Raisins	Peanuts
AFB1								
ML	30.8 (8.1)	**97.8 (0.6)**	94.4 (1.9)	96.4 (2.8)	78.9 (9.2)	93.9 (2.5)	**95.4 (2.6)**	89.8 (3.8)
5 × ML	33.1 (2.5)	**99.2 (0.6)**	79.1 (0.8)	98.9 (0.3)	73.4 (1.1)	88.1 (1.8)	**79.3 (0.5)**	93.8 (1.8)
10 × ML	23.1 (2.4)	**99.2 (0.4)**	82.7 (1.7)	92.7 (0.5)	65.1 (0.7)	83.1 (0.7)	**88.3 (0.8)**	85.4 (0.7)
AFB2								
ML	5.9 (9.4)	**98.8 (0.4)**	35.4 (1.1)	98.6 (1.0)	38.7 (15.2)	92.2 (1.7)	**85.3 (0.6)**	92.6 (1.2)
5 × ML	4.6 (2.5)	**98.9 (0.5)**	42 (1.1)	98.8 (0.5)	15.5 (0.8)	88.6 (0.5)	**80.0 (0.7)**	93.9 (0.2)
10 × ML	3.1 (3.2)	**92.6 (0.4)**	26.1 (1.0)	85.6 (0.5)	12.1 (1.4)	82.6 (0.3)	**80.4 (0.5)**	85.2 (0.3)
AFG1								
ML	9.9 (19.9)	**94.5 (5.2)**	70.4 (5.2)	94.2 (3.5)	67.5 (4.8)	92.0 (5.4)	**89.3 (2.6)**	86.8 (4.4)
5 × ML	11.2 (13.4)	**98.9 (0.7)**	67.9 (3.5)	98.1 (1.0)	43.9 (3.2)	89.9 (1.8)	**78.7 (1.7)**	96.4 (1.8)
10 × ML	7.6 (15.8)	**97.9 (0.9)**	53.8 (1.5)	91.4 (1.2)	36 (2.7)	83.2 (0.9)	**84.6 (0.9)**	85.9 (1.0)
AFG2								
ML	1.1 (5.3)	**98.3 (2.3)**	13.9 (1.8)	97.7 (1.3)	17.1 (4.5)	93.1 (1.6)	**82.6 (0.8)**	92.5 (1.4)
5 × ML	2.6 (5.4)	**92.7 (0.3)**	16.2 (0.7)	95.4 (0.2)	5.4 (1.7)	88.6 (0.3)	**72.4 (0.3)**	93.7(0.4)
10 × ML	1.6 (5.4)	**85.7 (0.2)**	8.2 (0.8)	78.2 (0.4)	4.1 (1.9)	82.2 (0.4)	**73.8 (0.3)**	85.3 (0.4)

*Note*: Bold indicates that these obtained recoveries values were good in accordance with requirements.

### 3.3 Validation results

#### 3.3.1 Influence of sample matrices

The calibration curve was performed on the solvent and the raisin and peanut matrices to evaluate the percent of the matrix effect (%ME) based on the coefficient of the standard curve equation (Chawla et al., 2017):

$$\%\mathrm{ME}=\left({\text{Slope}}_{\text{matrix}}/{\text{Slope}}_{\text{solvent}}–1\right)\times 100$$

A matrix effect is considered minimum, medium, or maximum, if the absolute matrix effect is ≤20%, 20%–50%, >50%, respectively. Table 4 shows the standard curve equations obtained for AFB1, AFB2, AFG1, and AFG2 dependent on solvent and matrix. The calculated |%ME| values were from 2.8% to 10.1% lower than 20% for all compounds. It indicates that the sample treatment process was almost not affected by the matrix. So, the calibration curve in solvent can employ to validate this procedure.

**TABLE 4:** The results % ME from the calibration curve on solvent and peanut and raisin matrices.

Compounds	Solvent	Raisin matrix		Peanut matrix	
	Curve	Curve	%ME	Curve	%ME
Aflatoxin B1	*Y* = 1.38e + 005*X*−3.04e + 003	*Y* = 1.24e + 005*X*−5.56e + 003	10.14	*Y* = 1.27e + 005*X*−5.41e + 003	7.97
Aflatoxin B2	*Y* = 1.31e + 006*X*−1.34e + 004	*Y* = 1.20e + 006*X* + 3.06e + 003	8.40	*Y* = 1.24e + 006*X*−8.84e + 003	5.34
Aflatoxin G1	*Y* = 6.75e + 004*X* + 8.32e + 003	*Y* = 6.37e + 004*X* + 7.03e + 003	5.63	*Y* = 6.56e + 004*X*−1.92E + 003	2.81
Aflatoxin G2	*Y* = 1.12e + 006*X* −4.74E + 003	*Y* = 9.92e + 005*X*−3.34e + 003	11.43	*Y* = 1.03e + 006*X*−2.06e + 004	8.04

The results of Table 4 show that the % ME in the raisin matrix is higher than in the peanuts, which shows that raisins contain more carbohydrates that affect the extraction of aflatoxin in the sample.

#### 3.3.2 Calibration curves

**FIGURE 3:**
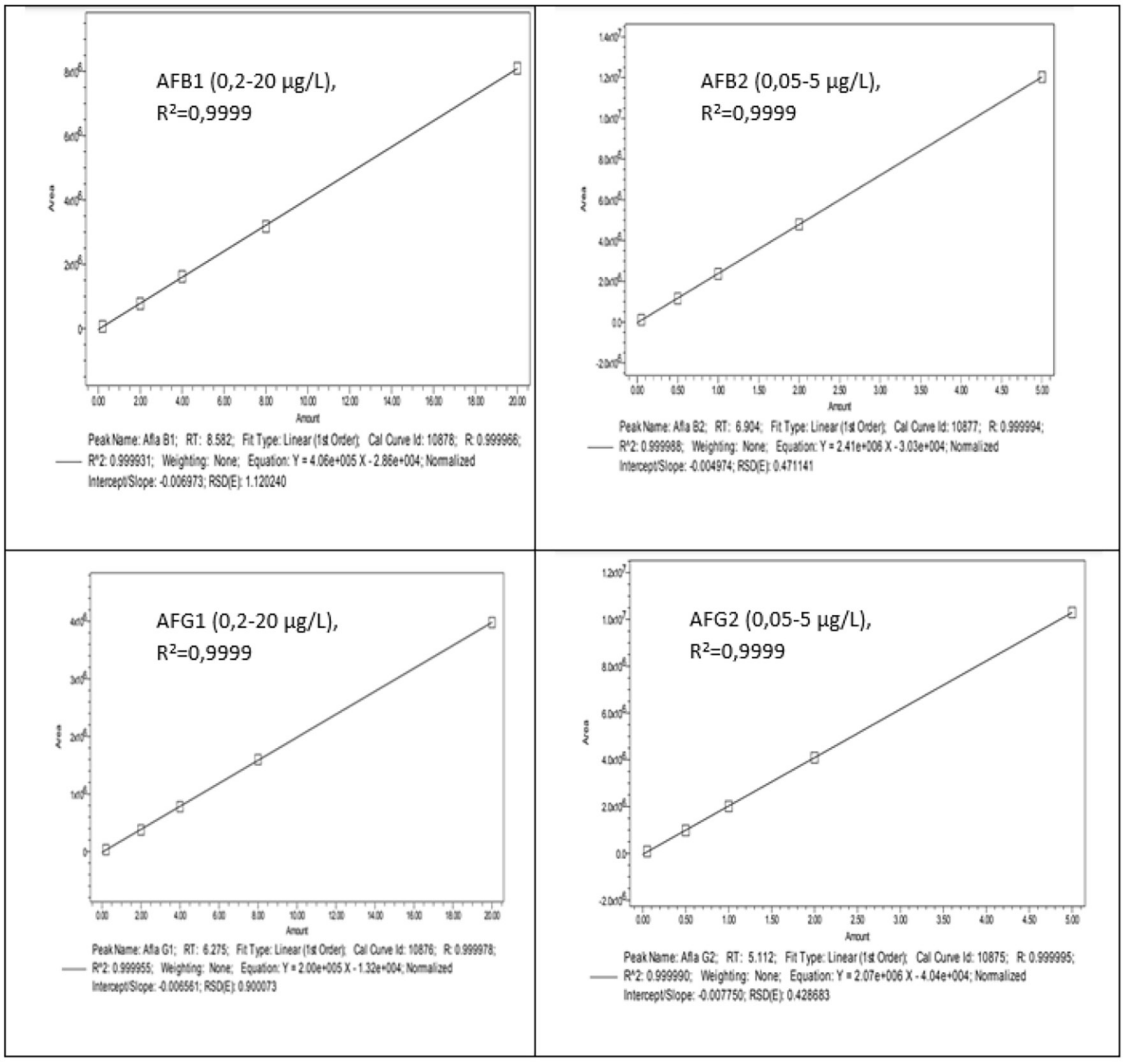
Standard curve of aflatoxins solution 0.05–20.0 μg/L.

Calibration curves were based on five points with concentrations in the 0.05–20 μg/L range. The coefficient of squared regression of calibration curves (*R* ^2^) was higher than 0.99, which satisfied the requirement in the linear concentration range (Figure 3).

The results of the linear range of the standard curve cover the concentration levels from LOQ up to the allowable limit on both matrices. On the other hand, the average calibration concentrations should be within 15% of the nominal concentration at all different concentrations. That is suitable for calibration curves used for quantification according to VICH GL49(R) (Veterinary International Conference on Harmonization Guidance Documents VICH GL49(R), 2016).

#### 3.3.3 System suitability

**FIGURE 4:**
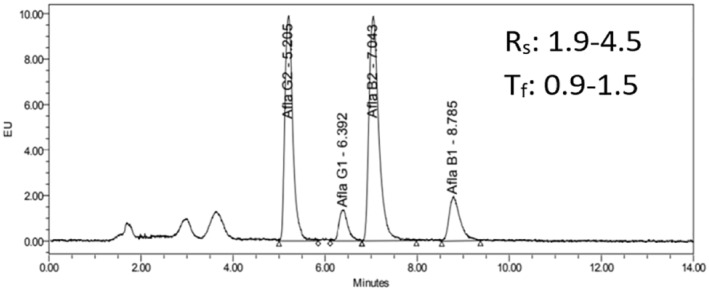
Standard chromatogram 10 μg/L with mobile phase Mp 3.

Use a standard solution to mix aflatoxin 10 μg/L in ACN/HCOOH 0.2%. Change three different mobile phase conditions as Mp 1—H_2_O/ACN/MeOH (56/22/22, v/v/v), Mp 2—H_2_O/ACN/MeOH (64/18/18, v/v/v), and Mp 3—0.1% HCOOH/ACN/MeOH (64/18/18, v/v/v). System suitability was assessed based on resolution (*R*_s_) and tail drag coefficient (*T*_f_), which require *R*_s_ ≥ 2 and 0.8 ≤ *T*_f_ ≤1.5 (Tome et al., 2019). The obtained results are presented in Table 5. *R*_s_, *T*_f_ values obtained from mobile phase Mp 2 and Mp 3 showed that both 2 and Mp 3 satisfied the requirements. Resolution and tailing factors were calculated using chromatographic data from Waters Empower 3 Software. The mobile phase composition mainly influences the peak response and retention of analytes. In addition, formic acid is a common additive component of mobile phases when using reversed‐phase liquid chromatography separations. The presence of a low concentration of formic acid in the mobile phase is also known to improve the peak shapes of the resulting separation (Núñez & Lucci, 2014). In this case, the most optimum mobile phase Mp 3 was chosen because of its good peak area and shape for simultaneous analysis of AFB1, AFB2, AFG1, and AFG2 in the matrices. The standard chromatogram at 10 μg/L of aflatoxins with mobile phase Mp 3 is presented in Figure 4.

**TABLE 5:** Resolution and tailing coefficients of aflatoxin peaks at 10 μg/L.

Compound	Mp 1 (*n* = 3)		Mp 2 (*n* = 3)		Mp 3 (*n* = 3)	
	*R*_s_	*T*_f_	*R*_s_	*T*_f_	*R*_s_	*T*_f_
AFB1	3.0	1.6	4.4	1.5	4.5	1.5
AFB2	1.7	1.6	1.8	1.5	1.9	1.5
AFG1	2.5	1.5	3.7	1.2	3.8	0.9
AFG2		1.7		1.4		1.4

#### 3.3.4 Selectivity

**FIGURE 5:**
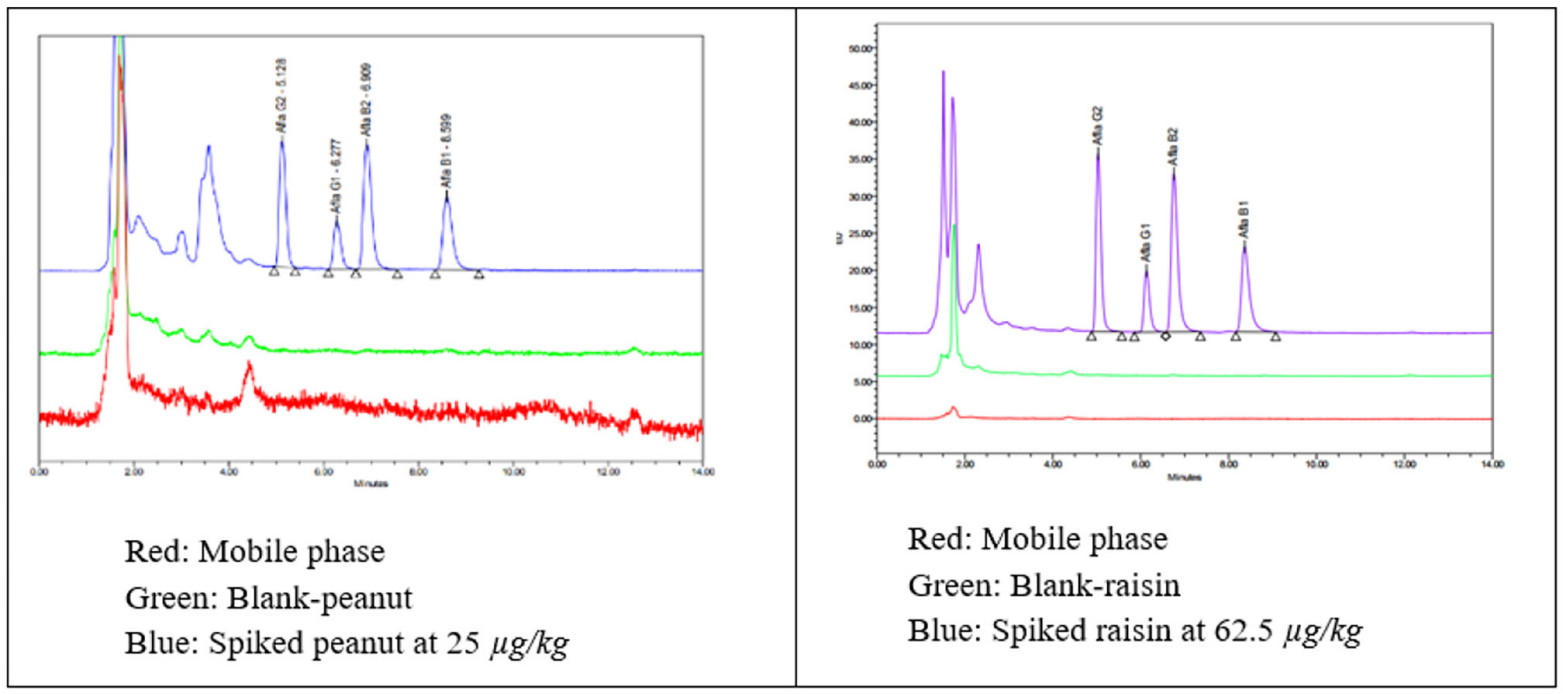
Overlay chromatograms of mobile phase, blank‐sample, spiked sample at 25 μg/kg for peanut (left) and 62.5 μg/kg for raisin (right).

The chromatogram of mobile phase and blank samples did not show any signals at the positions of AFB1, AFB2, AFG1, or AFG2 in comparison with the chromatograms of standards (Figure 5). Thus, there were not any background influences that indicate a good selectivity.

#### 3.3.5 Carryover

**FIGURE 6:**
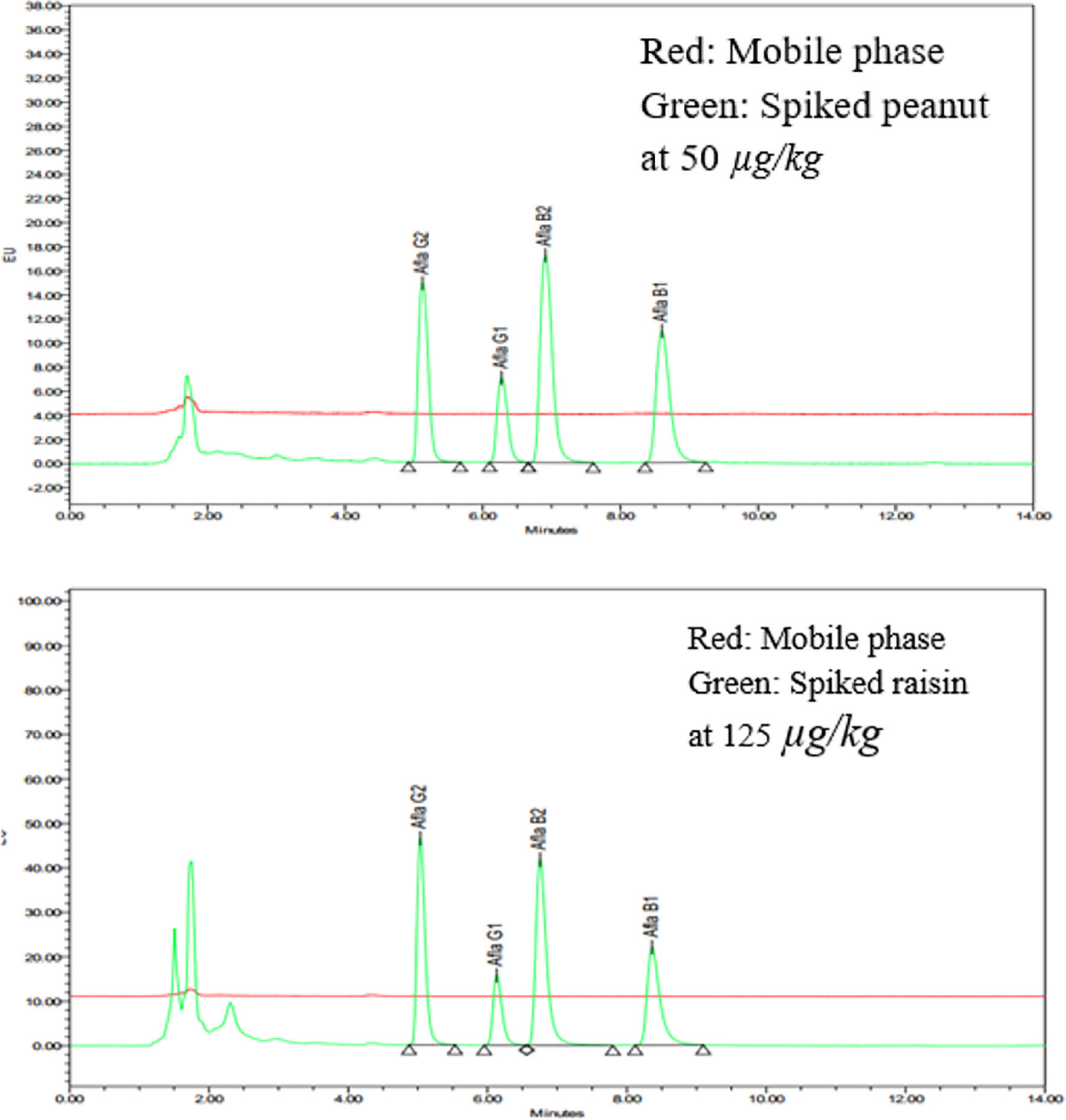
Solvent chromatograms of mobile phase and spiked sample at 50 μg/kg for peanut (left) and 125 μg/kg for raisin (right).

Figure 6 presents the chromatograms of the sample spiked at a high concentration (50 μg/kg for peanut, 125 μg/kg for raisin) in comparison with the chromatogram of the solvent after injection of the spiked sample. There were not any traces of aflatoxins which have been detected in the chromatogram of the solvent. Therefore, it indicates that the method satisfied the requirement of carrying over.

#### 3.3.6 Accuracy

##### The raisin matrix

Prepare separately three spiked samples (QCs) with 12 replicates of each sample. The final concentrations of four samples were 0.75 μg/kg (LOQ), 12.5 μg/kg (1 × ML), 62.5 μg/kg (5 ×ML), and 125 μg/kg (10 ×ML). Analyze samples with the investigated method and calculate the recovery (%H) of each QC sample. The obtained results are presented in Table 6a.

**TABLE 6:** The recovery, repeatability, and within‐laboratory reproducibility for the determination of aflatoxins in (a) spiked raisins from 0.75 to 125 μg/kg and (b) spiked peanut from 0.75 to 50 μg/kg.

Compound	Spiked sample at 0.75 μg/kg AFB1: 0.3 μg/kg, AFB2: 0.075 μg/kg AFG1: 0.3 μg/kg, AFG2: 0,075 μg/kg			Spiked sample at 12.5 μg/kg AFB1: 5 μg/kg, AFB2: 1.25 μg/kg AFG1: 5 μg/kg, AFG2: 1.25 μg/kg			Spiked sample at 62.5 μg/kg AFB1: 25 μg/kg, AFB2: 6.25 μg/kg AFG1: 25 μg/kg, AFG2: 6.25 μg/kg			Spiked sample at 125 μg/kg AFB1: 50 μg/kg, AFB2: 12.5 μg/kg AFG1: 50 μg/kg, AFG2: 12.5 μg/kg		
AFB1	% RSDr (*n* = 6)	% RSDR (*n* = 12)	% H (*n* = 12)	% RSDr (*n* = 6)	% RSDR (*n* = 12)	% H (*n* = 12)	% RSDr (*n* = 6)	% RSDR (*n* = 12)	% H (*n* = 12)	% RSDr (*n* = 6)	% RSDR (*n* = 12)	% H (*n* = 12)
(a)												
AFB2	1.9	1.9	86.4	0.3	0.5	97.3	0.3	0.5	92.8	0.2	0.6	89.3
AFG1	1.1	1.1	96.9	0.6	1.1	94.7	0.1	0.6	92.8	0.4	0.8	91.5
AFG2	2.1	2.3	91.3	0.7	0.9	97.1	0.3	0.7	90.8	0.3	0.8	86.9
AFB1	1.8	1.8	95.0	0.3	0.6	80.9	0.2	0.9	90.7	0.4	0.7	88.9
(b)												
AFB2	1.4	1.7	97.3	1.1	1.1	98.1	0.2	0.3	94.9	0.8	1.8	97.3
AFG1	1.7	1.7	94.9	0.2	0.8	98.6	0.2	0.3	91.7	0.9	3.9	92.3
AFG2	1.9	2.1	96.7	0.4	0.9	99.8	0.2	0.3	95.5	0.6	1.5	96.9
AFB1	1.1	1.2	99.4	0.2	0.2	96.0	0.1	0.5	84.6	0.6	3.0	76.5

##### The peanut matrix

Prepare three spiked samples (QCs) separately with 12 replicates of each sample. The final concentrations of four samples are 0.75 μg/kg (LOQ), 5.0 μg/kg (1 × ML), 25.0 μg/kg (5 × ML), and 50.0 μg/kg (10 × ML). Analyze samples with the developed method and calculate the recovery (%H) of each QC. The results are shown in Table 6b.

#### 3.3.7 Precision (repeatability—% RSD_r_ and reproducibility—% RSD_R_)

The preparation of spiked samples for precision assessment is similar to the accuracy assessment. QC samples were performed with six replicates for each concentration level, two persons prepared on two different days. The results of recovery, repeatability, and reproducibility (Table 6a,b) were following the AOAC Official Methods of Analysis (2019) (SANTE 11312/2021, 2021).

#### 3.3.8 The limit of detection and limit of quantification

According to (SANTE 11312/2021, 2021), the limit of quantification (LOQ) is the lowest spiked level of the validation for meeting these method performance acceptability criteria. Mean recoveries from initial validation should be within the 70%–120% range, with an associated repeatability RSDr ≤20%. This study prepared spiked samples at a concentration of 0.75 μg/kg for peanut and raisin samples to determine the LOQ value. Analytical results were obtained for all AFB1, AFB2, AFG1, and AFG2, with recovery over 70% and reproducibility less than 20%.

The spiked sample performed limit of detection (LOD) at a concentration that was lower three times than the LOQ value to determine the S/N ratio. The obtained results showed that the LOD and LOQ of this method for peanut and raisin matrices were in the range of 0.025–0.1 and 0.075–0.3 μg/kg, respectively. Whereby, LODs were 0.1, 0.025, 0.1, 0.05 μg/kg and LOQs were 0.3, 0.075, 0.3, 0.075 μg/kg for AFB1, AFB2, AFG1, and AFG2, respectively.

#### 3.3.9 Sample stability

Prepare two spiked samples (QCs) separately with three replicates of each level. The final concentrations of two QCs were 5 μg/kg, 50 μg/kg for peanut matrix, and 12.5 μg/kg, 125 μg/kg for raisin matrix. The samples were stored under different conditions as below:

• Spiked samples were extracted by the developed method. First, the extracts were stored in the autosampler at 20°C and 4°C for 24 h. Then, they were directly injected into the UPLC.
• Spiked samples not processed, were stored at −20°C for 10 and 21 days. The samples were extracted, and the extracts were injected into the UPLC.

**FIGURE 7:**
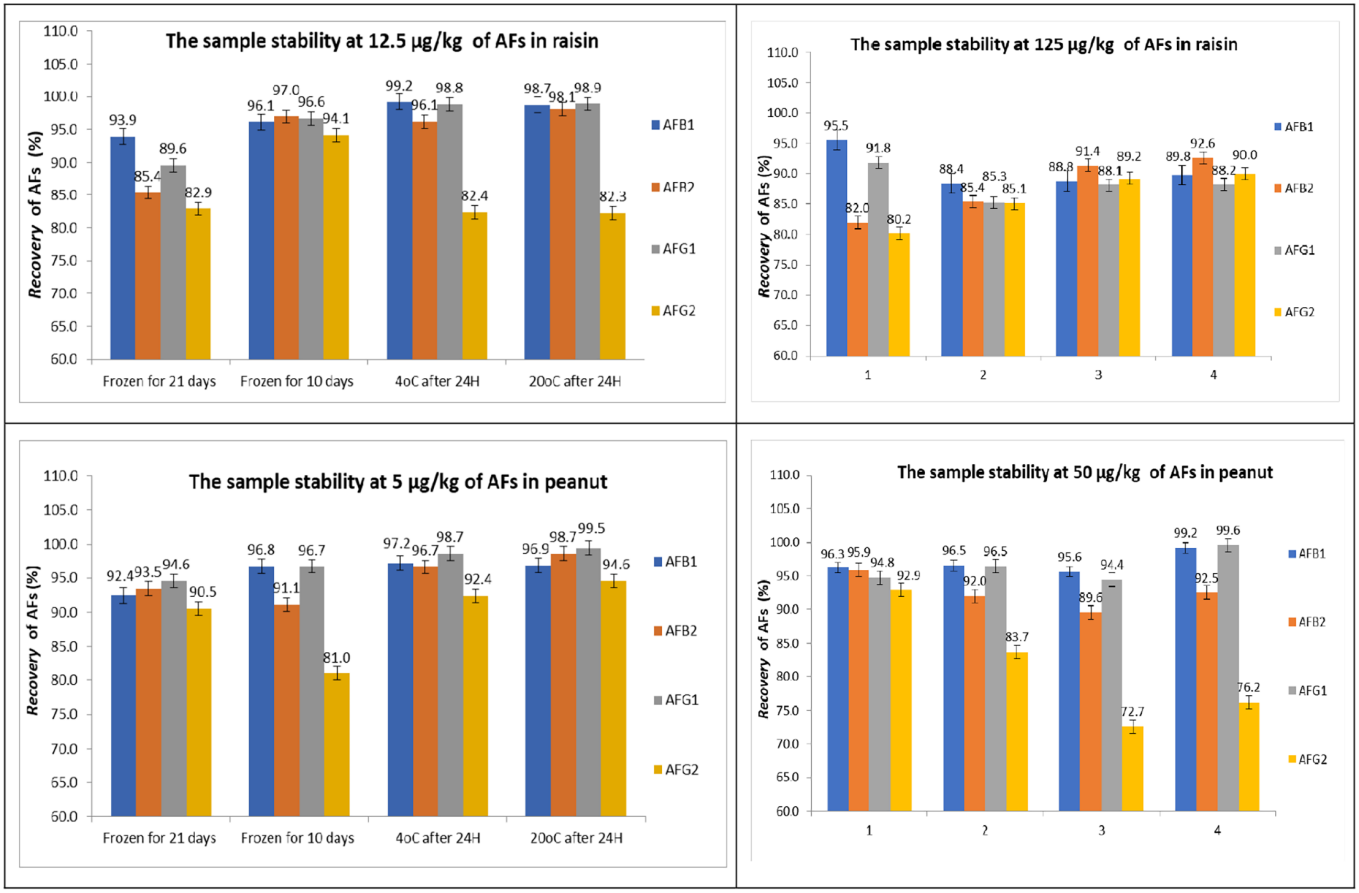
Bar charts of sample stability in raisin and peanut matrices under different storage conditions.

The recoveries for raisin and peanut matrices were 80.2%–98.7% at concentrations 12.5 and 125 μg/kg and 76.3%–99.6% at concentrations 25 and 50 μg/kg, respectively (Figure 7). This shows that the samples were almost unchanged and aflatoxins did not proliferate under storage conditions.

### 3.4 Survey results on peanut and raisin samples collected from markets at HCM city in Vietnam

The below formula was applied to calculate the size of survey samples (Hoang & Luu, 2020; Taherdoost, 2016):

$$n=\frac{z^{2}\times p.\left(1-p\right)}{e^{2}}$$

where *n* is the minimum number of samples calculated by the formula; *z* the distribution value corresponding to the 95% confidence level, looking up the table *z* = 1.96. *p* the estimated rate of food group with AF detected. *e* the desired error, chosen as 0.05.

The estimated percentage of peanuts and peanut products (52.4%) is referenced according to the data of several studies in Vietnam (81.3%; Nguyen, 2013), 23.6% (Do et al., 2020) corresponding to the expected sample size being 383 samples. For raisin matrix, to our knowledge, there is no domestic reference data. So, a random value of 50% was chosen, corresponding to an expected sample size being 384 samples.

In this study, 350 samples of peanuts and peanut products and 350 samples of raisins were collected at some markets in the central districts of Ho Chi Minh City. The obtained result is shown in Table 7.

**TABLE 7:** Survey results of aflatoxin content in peanuts and raisins in central districts of Ho Chi Minh City.

Matrix	Contaminated samples with AFB1 (%)	Contaminated samples with AFB2 (%)	Contaminated samples with AFG1 (%)	Contaminated samples with AFG2 (%)	Exceeding for AFB1 (%)	Exceeding limit for AF Total (%)
Peanuts and peanut products (*n* = 350)	100 (0.31–554 μg/kg) (28.6)	47 (0.33–110 μg/kg) (13.4)	20 (0.4–7.06 μg/kg) (5.7)	2 (0.45–0.75 μg/kg) (0.6)	47 (13.4)	45 (12.8)
Raisins (*n* = 350)	3 (0.82–1.48 μg/kg) (0.8)	0	0	0	0	0

For peanuts and peanut products, among 350 samples collected at Ho Chi Minh City markets, there were 100 samples which were contaminated with AFB1 with concentrations in the range of 0.31–554 μg/kg, accounting for 28.6% of the samples. Besides, AFB2 was detected in 47 samples (0.33–110 μg/kg, 13.4%); 20 samples contained AFG1 (0.4–7.06 μg/kg, 5.7%). And two samples were detected AFG2 (0.45–0.75 μg/kg, 0.6%). 250 samples were found not contained AFB1, AFB2, AFG1, AFG2, or below the detection limit (about 71.4%). In considering the total aflatoxins contamination, there were 45 samples (about 12.8%) exceeded 0.08 up to 167 times the allowable aflatoxin concentration (4 μg/kg). And for the samples contaminated with AFB1, 47 samples (about 13.4%) were found exceeded 1.03–277 times the maximum allowable amount (2 μg/kg) according to QCVN 8‐1:2011/BYT Vietnam. The rate of samples contaminated with AF in this study (28.6%) is also similar to some previous studies, such as author (Lien et al., 2019), which surveyed 1089 samples of peanuts imported from Taiwan and found a sample rate of 25%, with 3.1% of samples exceeding the allowable limit for total AF (ML = 4 μg/kg), and (Do et al., 2020), which surveyed 144 peanut samples in the northern provinces of Vietnam and found a contamination rate of 23.6%.

For the 350 raisin samples, only three samples were detected with AFB1 in the range of 0.82–1.48 μg/kg, which is lower than the maximum allowable limit for AFB1 according to QCVN 8‐1:2011/BYT Vietnam (ML = 5 μg/kg). None of the remaining samples were found to contain AFB1, AFB2, AFG1, or AFG2. The obtained results are in agreement with previous studies, which found no samples exceeding the allowable levels of total AF (ML = 10 μg/kg) and aflatoxin B1 (ML = 5 μg/kg), as follows:

Author (Asghar et al., 2016) surveyed 170 samples of raisins for export in Pakistan, of which only 5% of the total samples were contaminated with AF, ranging from 0.15 to 2.58 μg/kg, and no samples exceeded the allowable threshold. Yilmaz (2017) surveyed 25 seedless black raisin samples in Turkey, with the results showing that 16 out of 25 samples were detected with total aflatoxin concentrations ranging from 0.02 to 2.07 μg/kg, and no samples exceeded the allowable threshold.

Raisins are one of the imported and exported foods with high value, so technologies such as drying, packaging, and preservation are focused on significantly reducing the generation of AF toxins. One of these methods is ozone treatment before grape drying, which is said to be quite advantageous in terms of drying time and final product quality, and effective for reducing mold and yeast growth, especially during long‐term storage of raisins. Another method is sulfuring, in which sulfur dioxide (SO_2_) is used to prevent the development of microorganisms and preserve the color and flavor of foods, especially raisins (Aktas, 2015). In addition, the carbohydrate‐rich raisins can be coated to prevent water from entering, and the semi‐closed storage method creates an oxygen‐poor, carbon dioxide‐rich environment. Such conditions are unfavorable for the growth and production of AF by strictly aerobic AF molds (Naeem et al., 2022).

Peanuts are a very popular food in Vietnamese family meals. These crunchy, sweet, and easy‐to‐eat nuts are packed with nutrients and have many health benefits. They can be used to make many delicious dishes and incredibly tasty snacks. In addition, peanut by‐products such as stems, leaves, and shells contain a high‐protein content suitable for cattle feed. Aflatoxin M1 (AFM1), a derivative of AFB1, occurs in dairy cows fed an AFB1‐contaminated diet and can subsequently be transferred to other dairy products. AFB1 and AFM1 are hepatotoxic and carcinogenic, classified by the International Agency for Research on Cancer (IARC) of the World Health Organization (IARC, 2002) as Group 1 human carcinogens (Xiong et al., 2015). With a high concentration of protein and triglycerides, if peanuts are not stored in good conditions, they can create a favorable environment for the growth of Aspergillus fungi. Therefore, consumption of peanuts contaminated with AF directly or indirectly through livestock products such as milk and meat that are also contaminated with AF can be dangerous to humans.

## 4 CONCLUSION

The simultaneous quantitative procedures for four types of aflatoxins (AFB1, AFB2, AFG1, AFG2) were fully validated for peanut and raisin matrices. Furthermore, the obtained parameters were in accordance with the requirements of ISO/IEC 17025:2017, indicating that the methods have high accuracy and reliability for analyzing aflatoxins in peanut and raisin matrices.

The survey results on peanut and raisin matrices in the local Ho Chi Minh City showed that high levels of aflatoxin contamination were detected in the peanut matrix, while the contamination was negligible in the raisin matrix, which has a high carbohydrate content.

## AUTHOR CONTRIBUTIONS

**Duy Thanh Nguyen:** Data curation (lead); formal analysis (lead); investigation (lead); methodology (lead); project administration (lead); resources (lead); validation (lead); visualization (lead); writing – original draft (lead); writing – review and editing (lead). **Ha Thuy Ngan Nguyen:** Investigation (supporting); methodology (supporting); resources (equal); validation (supporting). **Kiet Tuan Ly:** Methodology (supporting); project administration (supporting); resources (supporting). **Tho Thanh Le:** Funding acquisition (supporting); methodology (supporting); project administration (supporting); resources (supporting). **Hung Quoc Nguyen:** Data curation (equal); investigation (equal); methodology (equal); supervision (lead); validation (equal); visualization (equal); writing – original draft (equal); writing – review and editing (equal). **Hai Van Chu:** Funding acquisition (supporting); methodology (supporting). **Dat Tien Nguyen:** Investigation (supporting); methodology (supporting); project administration (supporting); resources (equal); supervision (lead). **Vu Dinh Le:** Investigation (supporting); methodology (supporting); supervision (supporting); writing – review and editing (supporting).

## Data Availability

Data sharing is not applicable to this article as no new data were created or analyzed in this study.
